# Polymorphism of *splicing factor 3A subunit 3/PstI* gene and its association with the performance of Madura cows (*Bos indicus*)

**DOI:** 10.14202/vetworld.2025.1306-1312

**Published:** 2025-05-25

**Authors:** Widya Pintaka Bayu Putra, Hartati Hartati, Endang Tri Margawati, Mariyono Mariyono, Tulus Maulana, Thobela Louis Tyasi

**Affiliations:** 1 Research Center for Applied Zoology, National Research and Innovation Agency (BRIN), Bogor 16911, Indonesia; 2 Research Center for Animal Husbandry, National Research and Innovation Agency (BRIN), Bogor 16911, Indonesia; 3 Department of Agricultural Economics and Animal Production, School of Agricultural and Environmental Sciences, University of Limpopo, Private Bag X1106, Sovenga 0727, South Africa

**Keywords:** body measurements, genetic diversity, Madura cattle, marker-assisted selection, polymerase chain reaction-restriction fragment length polymorphism, *SF3A3* gene, single nucleotide polymorphism

## Abstract

**Background and Aim:**

Madura cattle (*Bos indicus*), a native Indonesian breed, are primarily raised for meat production and possess unique genetic characteristics shaped by crossbreeding with *Bos javanicus*, *Bos taurus*, and *B. indicus*. Despite their cultural and economic importance, limited molecular studies have explored candidate genes influencing their productive traits. This study aimed to identify the single-nucleotide polymorphism (SNP) g.1292A>T (rs473122879) in intron 2 of the *Splicing Factor 3A Subunit 3* (*SF3A3*) gene and to evaluate its association with key phenotypic traits in Madura cows.

**Materials and Methods:**

A total of 49 adult Madura cows (>3 years) were sampled from two breeding locations: Java (n = 29) and Madura Island (n = 20). Body weight, withers height, hip height, body length (BL), and heart girth were measured using standardized morphometric techniques. Genomic DNA was extracted from whole blood and subjected to polymerase chain reaction (PCR) amplification targeting a 633 bp fragment of the *SF3A3* gene. Genotyping of SNP g.1292A>T was performed using PCR-restriction fragment length polymorphism with *PstI* enzyme digestion. Sequencing analysis was conducted for genotype confirmation. Genetic diversity indices and Hardy–Weinberg equilibrium were assessed, and association between genotype and phenotypic traits was evaluated using a general linear model.

**Results:**

Two genotypes, TT (0.69) and AT (0.31), were identified; the AA genotype was absent in all samples. The polymorphism was under Hardy–Weinberg equilibrium (χ^2^ < 3.84) with a moderate polymorphic information content (PIC = 0.23). A significant association was found between *SF3A3*/*PstI* polymorphism and BL in cows from Madura Island (p < 0.05), with heterozygous (AT) individuals exhibiting superior morphometric traits compared to TT homozygotes.

**Conclusion:**

The *SF3A3*/*PstI* gene is polymorphic in Madura cows and exhibits moderate genetic diversity. The presence of the g.1292A>T SNP is significantly associated with BL, particularly in animals from their native breeding environment. These findings suggest the potential utility of *SF3A3*/*PstI* as a genetic marker in molecular selection strategies aimed at improving Madura cattle productivity.

## INTRODUCTION

Madura is a local beef cattle breed originating from Madura Island, Indonesia. This breed can attain a body weight of approximately 192.11 ± 63.71 kg in males and 190.57 ± 61.24 kg in females by 20 months of age [1]. Consequently, Madura cattle older than 24 months are capable of producing carcass weights of 125.33 ± 39.39 kg in males and 114.56 ± 17.35 kg in females [2]. Genetic introgression in Madura cattle has occurred from both *Bos indicus* and *Bos javanicus* lineages, as confirmed by mitochondrial DNA analysis [3]. However, Hartatik *et al*. [4] reported the presence of *Bos taurus* introgression in Madura bulls based on the *SRY* gene.

Previously, single-nucleotide polymorphisms (SNPs) in genes such as *Growth Hormone Receptor* (GHR), *Leptin* (LEP), and *Myostatin* (MSTN) were identified as potential genetic markers associated with economically important traits in Madura cattle [1, 5, 6]. More recently, a genome-wide association study (GWAS) identified an SNP, g.1172A>G (rs471103293), located in intron 2 of the *Splicing Factor 3A Subunit 3* (*SF3A3*) gene, which is associated with body weight in Madura cattle [7].

The *SF3A3* gene plays a vital role in pre-messenger RNA (mRNA) splicing and transcriptional regulation [8]. According to the reference sequence (GenBank: NC_037330.1), bovine *SF3A3* is located on BTA3 and spans a 26,317-bp region comprising 17 exons. To date, polymorphism in the bovine *SF3A3* gene has not been previously reported. However, mutations in the human *SF3A3* gene have been implicated in modulating the risk of ovarian and breast cancers [9, 10]. In the present study, the SNP g.1292A>T (rs473122879) in intron 2 of bovine *SF3A3* was identified through sequencing analysis and subsequently detected using the *PstI* restriction enzyme through polymerase chain reaction-restriction fragment length polymorphism (PCR-RFLP) methodology [11].

Although Madura cattle represent a valuable indigenous genetic resource in Indonesia, there remains limited molecular characterization of genes influencing their economically significant traits. Previous studies by Hartati and Putra [1], Kuswati *et al*. [5], and Novianti *et al*. [6] have focused primarily on polymorphisms in candidate genes such as *GHR, LEP*, and *MSTN*, which demonstrated associations with growth performance and body conformation traits. However, the functional implications of splicing factor genes, particularly *SF3A3*, have not been extensively explored in livestock populations, including Madura cattle. Moreover, while a GWAS recently identified a novel SNP (g.1172A>G) within *SF3A3* associated with body weight in Madura cattle [7], no studies to date have investigated the presence and phenotypic association of other *SF3A3* variants – such as g.1292A>T – in this population. The lack of information on intronic polymorphisms within *SF3A3* and their potential influence on morphometric and productive traits represents a significant gap in the molecular breeding knowledge of Madura cattle.

The objective of this study was to identify and characterize the SNP g.1292A>T (rs473122879) located in intron 2 of the bovine *SF3A*3 gene in Madura cows and to evaluate its association with phenotypic performance traits, including body weight, withers height (WH), hip height (HH), body length (BL), and heart girth (HG). By determining the genetic diversity and potential functional relevance of this SNP, the study aims to contribute foundational data for the development of marker-assisted selection (MAS) programs targeting genetic improvement in Madura cattle.

## MATERIALS AND METHODS

### Ethical approval

The assessment of Madura cows on Java Island was approved by the Animal Committee of the Indonesian Agency for Agricultural Research and Development (Certificate No: Balitbangtan/Lolitsapi/Rm/11/2021). Similarly, the assessment of Madura cows on Madura Island was approved by the Animal Ethics Committee of the National Research and Innovation Agency (BRIN) (Certificate No: 153/KE.02/SK/07/2023).

### Study period and location

The blood samples of Madura cows from Java and Madura Islands were collected during 22–26 March 2021 and 17–20 September 2024, respectively. While the DNA analysis for Madura cows from Java and Madura Islands was performed in the Molecular Laboratory of Loka Penelitian Sapi Potong Grati, East Java (7–11 June 2021) and the Integrated Laboratory (iLab) of BRIN, West Java (2–8 October 2024), respectively.

### Animals and research sites

**Figure 1:**
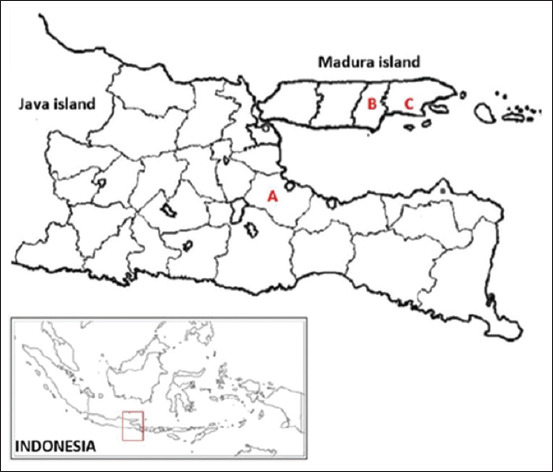
The research site to collect blood samples from Madura cattle at (A) Pasuruan Regency in Java island and (B) Pamekasan, and (C) Sumenep Regency in Madura island [Source: https://live.staticflickr.com/1885/43397567565_0d51a11767_b.jpg].

A total of 49 adult Madura cows (>3 years old) were selected from two breeding locations: Java (n = 29) and Madura Island (n = 20), as shown in Figure 1. A purposive sampling method was applied, selecting animals of the same age, breed, and sex under standard management conditions. Pasuruan Regency is geographically positioned between latitudes 7°32’34” to 7°57’20” S and longitudes 112°33’55” to 113°05’37” E, at an altitude of 0–12.5 m above sea level. The region experiences temperatures ranging from 20.6°C to 24.1°C, relative humidity of 60%–93%, and an annual rainfall of 3,072.3 mm. Madura Island is located at latitude 6°52’42” S and longitude 112°40’32” E, with an altitude of 6–312 m above sea level. It experiences temperatures of 21.0°C–29.0°C, relative humidity of 53%–92%, and annual rainfall between 1,328 mm and 1,571 mm.

### Animal management

Madura cows from Java Island were maintained at the Loka Penelitian Sapi Potong Grati breeding station (BSIP Ruminansia Besar). Natural mating was conducted with a ratio of 1 male to 20 females per stall. Each animal was fed 3–5 kg/day of forage (*Pennisetum purpureum* and *ad libitum* rice straw). The standard diet included 10%–11% crude protein, 58%–60% total digestible nutrients, and 17%–19% crude fiber. In addition, conce-ntrate feed comprising 3% of body weight was provided, including chalk (1.89%), salt (1.89%), rice bran (24.75%), slamper corn (20.51%), coffee peel (4.98%), palm kernel cake (10.16%), copra cake (10.16%), cassava flour (10.16%), distillers dried grains with solubles (7.98%), and corn gluten feed (7.98%).

Cows on Madura Island, typically selected for local contests, were housed individually at Villager Breeding Centers in Pamekasan and Sumenep Regencies. Artif-icial insemination was commonly used to increase calf production. The feed consisted of field and elephant grass (*P. purpureum*), rice or corn straw, and leaves of papaya (*Carica papaya*), bamboo (*Bambusa vulgaris*), mahogany (*Swietenia macrophylla*), leucaena (*Leucaena leucocephala*), mango (*Mangifera indica*), and banana (*Musa* spp.). Rice or corn bran was provided once daily. Feeding occurred 3 times/day, and cows received 20–40 L of water daily. Health monitoring and vaccinations were conducted alongside pregnancy examinations.

### Body weight and body measurements

**Figure 2:**
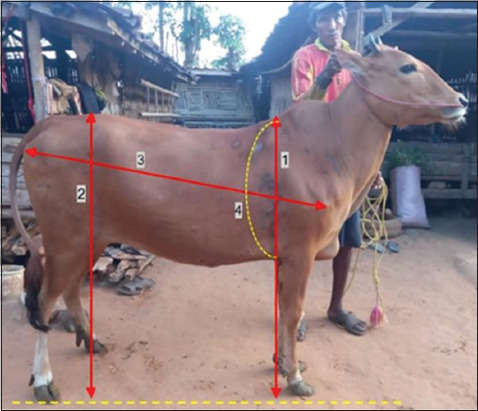
Scheme of body measurements in a Madura cow, i.e., (1) withers height, (2) hip height, (3) body length, and (4) heart girth.

Body weight was recorded using a digital scale (Sonic NI-7, China). Four body measurements – WH, HH, BL, and HG – were obtained following the method described by Sawanon *et al*. [12]. WH was measured using a stick rule from the platform surface to the dorsal point of the withers. HH was determined as the distance to the dorsal point of the spina iliaca. BL was measured from the humeral head to the posterior end using a tape. HG was determined as the chest circumference just behind the forelegs. A schematic of these measurements is presented in Figure 2.

### DNA extraction and amplification of SF3A3 gene

Approximately 3 mL of blood was collected from each animal using a venoject needle and ethylene-diaminetetraacetic acid-coated vacutainer tubes (BD Vacutainer, USA) and stored at −20°C. A total of 100 μL of DNA was extracted using a Geneaid kit (Taiwan) per the manufacturer’s protocol. DNA concentration was standardized to 50 ng/μL, with purity (A260/A280) between 1.8 and 2.0. PCR amplification of a 633 bp fragment of the *SF3A3* gene (GenBank: NC_007301.5) was carried out using primers described by Putra [11]: forward 5′-CCT CCT CCC CCA TAG AAA AG-3′ and reverse 5′-CCA TGG ACA GAG GAG CCT AA-3′. PCR was performed in a total volume of 10 μL, comprising 1.5 μL DNA, 0.2 μL each primer (10 pmol), 5 μL MyTaq HS red mix (Bioline, USA), and 3.1 μL nuclease-free water. The thermal cycling conditions included initial denaturation at 94°C for 2 min, followed by 35 cycles of denaturation at 94°C for 90 s, annealing at 55.4°C for 1 min, extension at 72°C for 1 min, and final extension at 72°C for 5 min.

### Genotyping

SNP g.1292A>T (rs473122879) in intron 2 of the *SF3A3* gene was detected using the PCR-RFLP method. A 10 μL digestion mix included 4.2 μL PCR product, 0.28 μL *PstI* restriction enzyme (CTGCA*G), 0.70 μL buffer, and 1.82 μL nuclease-free water. DNA fragments were visualized on 2% agarose gel containing 3 μL FloroSafe DNA stain and a 100 bp DNA ladder (Invitrogen, USA) and imaged using a G-box documentation system (UVITEC, UK). Three genotypes were observed AA (508 bp and 125 bp), TT (633 bp), and AT (633 bp, 508 bp, and 125 bp).

### Sequencing

Sequencing was conducted to confirm the mutation site. A total of 25 μL PCR product (one sample per genotype) was sent to Apical Scientific Laboratory Services (Malaysia). The SNP site was analyzed using the BioEdit software package (BioEdit 7.2) [13].

### Statistical analysis

Genetic diversity parameters – including gen-otype and allele frequencies, observed and expected heterozygosity (Ho, He), effective allele number (ne), polymorphic information content (PIC), and Chi-square (χ^2^) values were calculated according to Nei and Kumar [14]. Associations between *SF3A3* genotypes and phenotypic traits were analyzed using a general linear model in the Statistical Package for the Social Sciences 16.0 (IBM Corp., NY, USA), based on the model:

Y_ijk_ = μ + G_i_ + E_ijk_

Where, Y_ij_ is the observed traits; μ is the common mean; G_i_ is the effect of i^th^ genotype, and E_ijk_ is the experimental error.

## RESULTS

**Figure 3:**
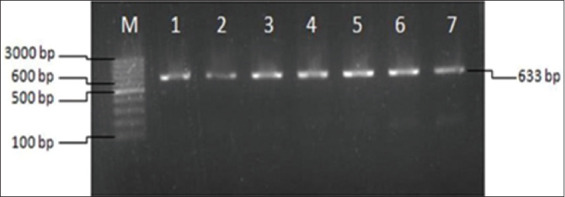
Amplification of the *SF3A3* gene in Madura cows at 633 bp on 1% agarose gel. M: DNA ladder; lane 1–7: DNA sample.

**Figure 4:**
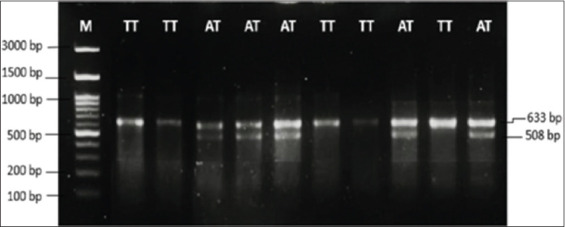
Pattern of PCR-RFLP results on 2% agarose gel. Two genotypes of TT (633 bp) and AT (633 bp and 508 bp) were observed in the *SF3A3*/*PstI* gene of Madura cows. The DNA fragment along 125 bp in AT genotype is not observed. M: DNA ladder of 100 bp.

**Figure 5:**
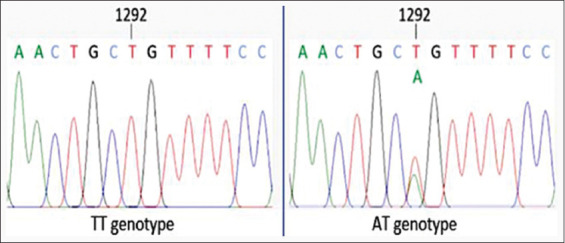
Detection of SNP g.1292A>T (rs473122879) in the intron 2 region of the bovine *SF3A3* gene (GenBank: NC_007301.5).

A 633 bp fragment of the partial *SF3A3* gene was successfully amplified and visualized on 1% agarose gel, as shown in Figure 3. Based on PCR-RFLP analysis (Figure 4), two genotypes – TT and AT – were identified in the *SF3A3*/*PstI* region of Madura cows. However, the AA genotype was not observed in any of the animals analyzed. Sequencing analysis confirmed a transversion mutation (A>T) at the 1292^nd^ nucleotide position of the bovine *SF3A3* gene (Figure 5).

In the Madura cattle population, the g.1292A>T SNP in *SF3A3* was polymorphic, with genotype frequencies of TT (0.69) and AT (0.31) (Table 1). The TT genotype was predominant in both subpopulations, comprising 62% of cows from Java and 80% from Madura Island. Moreover, the frequency of the T allele (0.85) was considerably higher than that of the A allele (0.15) across the pooled population.

**Table 1:** Genetic diversity in the *SF3A3/PstI* gene of Madura cows.

Parameter	Population (n)		Total (n = 49)

	Java (n = 29)	Madura (n = 20)	
Frequency of AA genotype	0.00 (0)	0.00 (0)	0.00 (0)
Frequency of the TT genotype	0.62 (18)	0.80 (16)	0.69 (34)
Frequency of the AT genotype	0.38 (11)	0.20 (4)	0.31 (15)
The frequency of the A allele	0.19	0.10	0.15
Frequency of the T allele	0.81	0.90	0.85
Expected heterozygosity (H_e_)	0.31	0.18	0.26
Observed heterozygosity (H_o_)	0.38	0.20	0.31
Number of effective alleles (n_e_)	1.44	1.22	1.23
Polymorphic informative content	0.26	0.16	0.23
Chi-square (χ^2^)	1.59*	0.25*	1.60*

n = number of animals,

* under genetic equilibrium, *SF3A3=Splicing factor 3A subunit 3*

Genetic diversity analysis revealed that the *SF3A3*/*PstI* locus in the pooled population had a PIC of 0.23 and an effective allele number (ne) of 1.23. Importantly, the population was found to be in Hardy–Weinberg equilibrium at this locus (χ^2^ < 3.84).

Preliminary analysis indicated a significant association between *SF3A3*/*PstI* polymorphism and BL in Madura cows, particularly those from their native breeding region (Table 2). Overall, heterozygous (AT) cows tended to exhibit greater body weights and body measurements compared to homozygous (TT) individuals.

**Table 2:** Effect of *SF3A3/PstI* polymorphism on the performance of Madura cows.

Population	Parameter	Genotype (n)	

		TT	AT
Java	Body weight (kg)	265.94 ± 39.55 (16)	280.75 ± 26.07 (8)
	Withers height (cm)	118.09 ± 4.95 (16)	118.12 ± 4.73 (8)
	Hip height (cm)	120.83 ± 3.40 (16)	120.72 ± 5.10 (8)
	Body length (cm)	120.18 ± 5.97 (16)	123.06 ± 4.94 (8)
	Heart girth (cm)	151.00 ± 7.58 (16)	150.12 ± 4.91 (8)
Madura	Withers height (cm)	130.81 ± 8.99 (16)	125.00 ± 8.44 (4)
	Hip height (cm)	130.12 ± 7.17 (16)	124.25 ± 4.79 (4)
	Body length (cm)	132.87 ± 8.61^a^ (16)	125.25 ± 15.30^b^ (4)
	Heart girth (cm)	164.91 ± 13.54 (16)	147.75 ± 17.99 (4)
Total	Withers height (cm)	124.45 ± 9.63 (32)	120.42 ± 6.72 (12)
	Hip height (cm)	125.48 ± 7.26 (32)	121.90 ± 5.08 (12)
	Body length (cm)	126.53 ± 9.73 (32)	123.79 ± 8.97 (12)
	Heart girth (cm)	157.95 ± 12.90 (32)	149.33 ± 10.24 (12)

n = number of animals, *SF3A3=Splicing factor 3A subunit 3,* ^a,b^Superscript in the different column differ significantly (p < 0.05)

## DISCUSSION

In general, the *SF3A3*/*PstI* gene in Madura cattle was polymorphic, exhibiting a moderate PIC value. According to Nei and Kumar [14], PIC values are categorized as low (<0.20), moderate (0.21–0.30), and high (>0.30). Several prior studies have reported high PIC values for candidate genes in Madura cattle, including *FTO*/HpyCH4III [15], *MC4R*/HpyCH4IV [16], *GHR*/AluI [17], and *LEP*/AciI [5]. Conversely, a low PIC value was reported for the *EDG1*/MscI gene in Madura cattle [18]. In addition, the *GH*/AluI and *MSTN*/DraI genes in Madura cattle have been reported as monomorphic [19–21]. Moreover, the *FSH*/*PstI* and *FSHR*/AluI genes were also found to be monomorphic in Limousin × Madura (Limura or Madrasin) crossbred cattle [20].

In the present study, the AA genotype was absent in the pooled Madura cattle population. This finding is similar to the results for the *FTO*/HpyCH4III gene, where the TT genotype was also absent [15]. The absence of the AA genotype in the *SF3A3*/*PstI* locus may be due to natural selection favoring animals better adapted to local environments, potentially rendering the AA genotype deleterious or even lethal in Madura cattle. The genetic diversity observed at the g.1292A>T site was in Hardy–Weinberg equilibrium, suggesting no recent selection pressure acting on this locus. Population genetic equilibrium can be influenced by factors such as migration, selection, crossbreeding, and inbreeding [22].

The polymorphism of *SF3A3*/*PstI* occurred within an intronic region. In eukaryotic organisms – including mammals, plants, yeasts, and insects – introns are known to enhance gene expression without necessarily serving as transcription factor binding sites. Moreover, introns can improve the efficiency of mRNA translation [23] and play essential roles in transcription initiation and termination [24].

A significant association was observed between *SF3A3*/*PstI* polymorphism and BL in Madura cows from Madura Island. Notably, in pooled animals, cows with the TT genotype exhibited higher body measurements than those with the AT genotype, aligning with the findings observed in the Madura Island subgroup. In this context, cows from Madura Island are often intensively reared by farmers for participation in traditional contests (Sonok culture), which may contribute to reduced phenotypic variability due to selective breeding. The low PIC value observed in this subpopulation could therefore reflect prior selection efforts.

As a preliminary investigation, the findings highlight the potential of *SF3A3*/*PstI* as a candidate genetic marker for inclusion in molecular breeding programs aimed at improving productive traits in Madura cattle. However, further studies involving larger sample sizes and more comprehensive phenotypic data are warranted to statistically validate the effects of *SF3A3*/*PstI* polymorphism on economically important traits in this breed.

## CONCLUSION

This study identified and characterized the g.1292A>T (rs473122879) SNP within intron 2 of the *SF3A3* gene in Madura cattle using PCR-RFLP and sequencing approaches. The *SF3A3*/*PstI* locus was found to be polymorphic, exhibiting two genotypes (TT and AT) with a moderate PIC (0.23), and was in Hardy–Weinberg equilibrium, indicating stable genetic variation. Notably, the TT genotype was predominant, while the AA genotype was absent across the studied population. A significant association was observed between the *SF3A3*/*PstI* polymorphism and BL, particularly in Madura cows from their indigenous breeding tract, suggesting a potential role of this intronic variant in modulating morphometric traits.

The strength of this study lies in its focus on an underexplored candidate gene involved in pre-mRNA splicing, which may indirectly influence phenotypic traits relevant to productivity. Moreover, the application of a dual-location sampling design enhanced the representativeness of the findings across different management and environmental conditions. The use of both PCR-RFLP and sequence confirmation further strengthens the reliability of genotype identification.

However, the study is not without limitations. The sample size was relatively modest (n = 49), and the absence of the AA genotype limits the ability to fully explore genotype-phenotype correlations. In addition, only a single SNP was investigated, and the influence of environmental or epigenetic factors on the observed traits was not assessed.

Future studies should aim to validate these findings in larger and more genetically diverse populations. Investigating the functional role of intronic SNPs in *SF3A3* through transcriptomic and gene expression analyses could provide mechanistic insights. Moreover, integrating this candidate gene into broader genomic selection or MAS programs may support the sustainable genetic improvement of Madura cattle, particularly for traits associated with body conformation and meat production potential.

## DATA AVAILABILITY

The data supporting the study findings are avail-able from the corresponding author on a reasonable request.

## AUTHORS’ CONTRIBUTIONS

WPBP and HH: Designed the study, collected data, performed fieldwork, supervised the study, and prepared and revised the manuscript. ETM, MM, TM, and TLT: Analyzed and interpreted the data and critically revised the manuscript for important intellectual content. All authors have reviewed and approved the final version.
